# GENet: A Graph-Based Model Leveraging Histone Marks and Transcription Factors for Enhanced Gene Expression Prediction

**DOI:** 10.3390/genes15070938

**Published:** 2024-07-18

**Authors:** Mahdieh Labani, Amin Beheshti, Tracey A. O’Brien

**Affiliations:** 1 School of Computing, Macquarie University, Sydney 2109, Australia; mahdieh.labani@hdr.mq.edu.au (M.L.); tracey.obrien@health.nsw.gov.au (T.A.O.); 2 Cancer Institute NSW, Sydney 2065, Australia; 3 School of Clinical Medicine, Medicine & Health, University of New South Wales (UNSW), Sydney 2052, Australia

**Keywords:** gene expression prediction, graph-based models, transcription factors, histone modifications, DNA sequence analysis, regulatory genomics

## Abstract

Understanding the regulatory mechanisms of gene expression is a crucial objective in genomics. Although the DNA sequence near the transcription start site (TSS) offers valuable insights, recent methods suggest that analyzing only the surrounding DNA may not suffice to accurately predict gene expression levels. We developed GENet (Gene Expression Network from Histone and Transcription Factor Integration), a novel approach that integrates essential regulatory signals from transcription factors and histone modifications into a graph-based model. GENet extends beyond simple DNA sequence analysis by incorporating additional layers of genetic control, which are vital for determining gene expression. Our method markedly enhances the prediction of mRNA levels compared to previous models that depend solely on DNA sequence data. The results underscore the significance of including comprehensive regulatory information in gene expression studies. GENet emerges as a promising tool for researchers, with potential applications extending from fundamental biological research to the development of medical therapies.

## 1. Introduction

Gene regulation is the process by which cells control gene expression, managing the production of proteins and other essential molecules necessary for life. This system ensures that genes are expressed at appropriate levels and times, responding to cellular and environmental changes. Various mechanisms are involved, including the use of DNA regions like promoters and enhancers, epigenetic changes that modify the structure of DNA and histones, and post-transcriptional mechanisms such as microRNAs. Understanding gene regulation is fundamental to bioinformatics and genomics, helping to decipher complex networks governing gene expression [1].

Multiple factors regulate genes at the DNA level, involving an interplay between genetic sequences and proteins. Transcription factors (TFs) play a pivotal role by binding to specific DNA sequences near genes, regulating transcription initiation and rate. Histone modifications, such as methylation and acetylation, influence gene expression by altering chromatin structure and accessibility. Non-coding RNAs, enhancers, silencers, and insulators also contribute to a dynamic regulatory network that maintains cellular homeostasis and adaptability [2].

Advancements in sequencing technology have enabled the quantification of gene expression and profiling of histone modifications and TF binding sites, providing a comprehensive view of the regulatory landscape. Initial studies, such as [3,4], investigated the correlation between histone modification marks and TFs. Despite their insights, TF and histone mark-based methods face challenges related to data interpretation, model complexity, and generalization capabilities. AI techniques and data integration strategies are crucial for overcoming these obstacles.

Histone modifications have been extensively studied, leading to the Histone Code Hypothesis, which posits that combinations of histone modifications determine chromatin states and gene regulation. Various methods, such as random forest (RF) [5], linear regression (LR) [6,7], rule-based learning [8], support vector machines (SVM) [9], and ReliefF [10], have been used to understand this relationship. These models face limitations, including dependency on feature selection and classification algorithms, overlooking minor signal variations, and failing to model connections between input bins.

Deep learning models have been proposed to address these problems by learning complex functions of histone marks and gene expression. For instance, DeepChrome [11] uses a convolutional neural network to predict gene expression from histone modification profiles. AttentiveChrome [12] employs a hierarchy of Long Short-Term Memory (LSTM) modules to explore relationships among chromatin factors. DeepDiff [13] predicts differential gene expression from histone modification signals using LSTM modules and attention mechanisms. Despite advancements, these models face challenges, including oversimplifying assumptions about TF binding and gene regulation [14,15], noise and inaccuracies in ChIP-seq data, ambiguous causality between histone marks and gene expression, and the need for context-specific models and interpretability.

Methods utilizing TFs for gene expression prediction include TEPIC [16], which integrates TF binding affinities with open chromatin data, and a study by [17] that uses ChIP-Seq to predict gene expression in embryonic stem cells. Despite their contributions, these methods also face challenges related to oversimplified assumptions about TF binding and gene regulation and inaccuracies in ChIP-seq data.

In this study, we leverage TFs and the histone modification H3K27ac to predict gene expression, drawing on their pivotal roles in gene regulation. This approach is grounded in their established influence over transcriptional activities. TFs initiate and control gene transcription, while H3K27ac marks active chromatin states conducive to transcription. Clinically, this model has profound implications for personalized medicine, particularly in oncology, where understanding and intervening in unusual gene expression can directly influence treatment outcomes. For instance, accurately predicting gene expression patterns allows for the identification of disease-specific regulatory mechanisms, potentially leading to targeted therapies and improved diagnostic precision. By incorporating this model, we aim to enhance our understanding of genetic regulation mechanisms, offering insights that could revolutionize treatment strategies and foster the development of precision medicine, thus addressing a critical gap in current medical research methodologies.

In this study, we specifically utilize H3K27ac, a histone modification known to mark active enhancers and promoters, as a crucial feature in our predictive model. H3K27ac is well recognized as a marker for active enhancers and an excellent indicator of enhancer activity, underscoring its reliability in identifying transcriptionally active regions [18]. This choice is pivotal for enhancing the accuracy of our gene expression predictions, given the modification’s direct association with transcriptional states across diverse cell types and conditions. We then combine these data with TF information to predict gene expression. Our approach utilizes graph convolutional networks (GCNs) for gene expression prediction. Compared to fully connected neural networks, GCNs use both the TF and histone features as well as the correlations among samples described by the similarity networks for better classification performance. Additionally, our method, GENet, employs a View Correlation Discovery Network (VCDN) to explore the feature correlations at the label space, effectively integrating histone and TF features. We demonstrate the capabilities and versatility of GENet across a wide range of cell lines.

The contributions of this work can be summarized as follows:

- We have developed a novel supervised model architecture that integrates TF binding sites and histone modification data, specifically H3K27ac marks. This integration is crucial for accurately predicting gene expression levels, harnessing both the regulatory and epigenetic landscapes.
- Our model uniquely applies GCNs to handle the classification task for each feature type. This choice uses the structural nature of genomic data, allowing the model to capture and utilize the complex relationships between different genomic features and their influence on gene expression.
- We construct weighted sample similarity networks using cosine similarity to quantify and utilize the relationships among samples. This network construction facilitates the effective handling of the spatial and functional relationships inherent in genomic data.
- GENet introduces a cross-feature discovery tensor that captures correlations between labels across different features. This innovative structure allows for the integration of insights across the genomic landscape, enhancing predictive accuracy.
- The culmination of our methodology involves transforming the discovery tensor into a vector that inputs into a regression model. This final step synthesizes all prior analyses to provide a comprehensive and refined prediction of gene expression levels.

## 2. Methodologies

### 2.1. Framework of GENet

**Figure 1:**
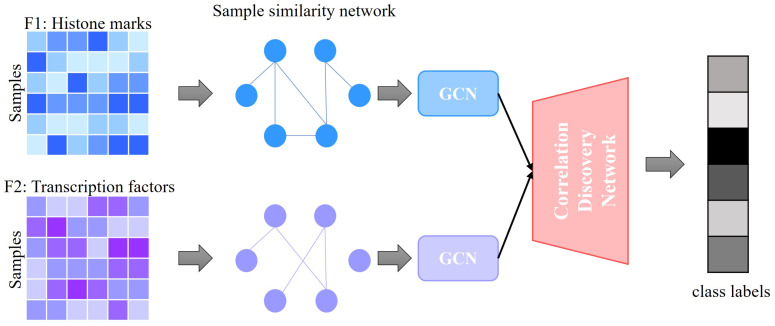
Workflow of GENet involving feature matrix construction, sample similarity network formation, GCN processing, and integration into the final prediction model. Each step is crucial in transforming raw data into actionable insights for gene expression prediction. This figure illustrates the process of integrating two distinct feature data types through GCNs. Each feature data type is first represented as a matrix. These matrices are then used to construct sample similarity networks, depicted as interconnected nodes. Separate GCNs process the networks to capture complex data relationships, outputting initial predictions. These predictions are subsequently fed into the Correlation Discovery Network, which merges the information to generate a final predictive output.

We have developed a novel supervised model architecture called GENet (Gene Expression Network from Histone and TF Integration) to predict gene expression, as illustrated in Figure 1. Initially, we employ GCNs to tackle the classification task for each feature. We create a weighted sample similarity network for each feature type using cosine similarity.

Each GCN receives similarity networks as inputs and is trained to produce preliminary class label predictions. A key strength of GCNs lies in their ability to harness both the attribute data and sample interconnections to enhance prediction accuracy. Subsequently, we use these initial predictions from each feature-specific GCN to assemble a cross-feature discovery tensor. This tensor identifies correlations between labels across different features. Ultimately, we transform this tensor into a vector, which is then applied to a regression model to make final label predictions.

### 2.2. Feature Matrix Construction

To investigate the regulatory mechanisms of gene expression, we constructed two types of feature matrices from the ENCODE dataset: one for TF binding sites and another for histone modifications, specifically H3K27ac marks. Our model is designed such that for each gene, the rows of these matrices correspond to various cell lines, while the columns represent discrete DNA positions within a defined window surrounding the TSS. This matrix structure allows for the comparison of epigenetic features across different cellular contexts and their positional influence relative to gene regulatory regions (Algorithm 1).
**Algorithm 1:** Construction of Cell Line- and Position-Specific Feature Matrices
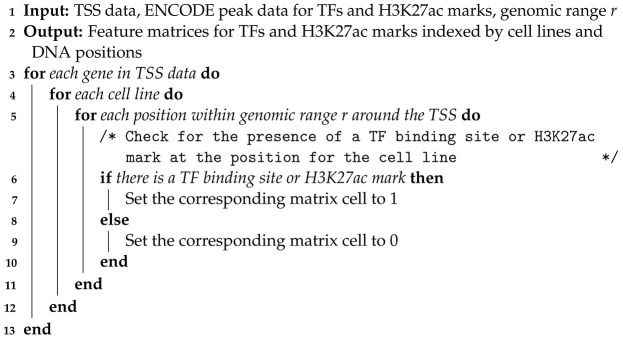


### 2.3. Weighted Sample Similarity Networks

We then proceed to construct weighted sample similarity networks for each data type by utilizing these feature matrices. This is achieved by calculating the cosine similarity between samples, which quantifies the similarity between two vectors in a multidimensional space; in this case, it represents the feature profiles of different samples. The outcome is a network where nodes correspond to samples, and edges represent the calculated similarities, weighted to reflect the degree of resemblance between the samples’ feature profiles (Algorithm 2).
**Algorithm 2:** Weighted sample similarity network construction
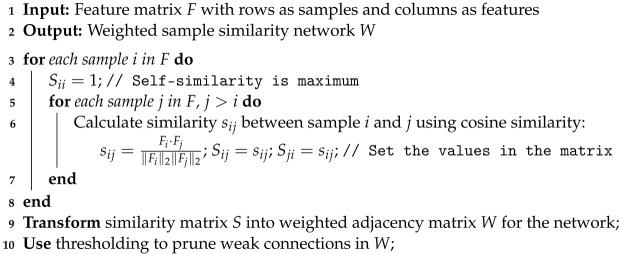


### 2.4. Training of Graph Convolutional Networks

Upon constructing the weighted sample similarity networks, we utilize Graph Convolutional Networks (GCNs) [19] to decode the complex data encoded within. A distinct GCN is dedicated to each type of data—TFs and H3K27ac marks—allowing for specialized learning from the respective similarity networks. This segmentation ensures that the unique properties and contributions of each data type are fully exploited.

GCNs are adept at processing graph-structured data, making them an ideal choice for our methodology. They extend the capabilities of traditional convolutional networks by applying convolution operations on graphs, thereby enabling the aggregation and processing of information from a node’s neighbors. Through this mechanism, GCNs can capture both local and global data structures, enriching the model’s understanding of sample relationships and feature interactions.

### 2.5. Integration and Final Prediction

The conclusion of our methodology is marked by the integration of initial predictions derived from each GCN. This integration is achieved through the construction of a cross-feature discovery tensor, a novel structure designed to encapsulate the inter-feature label correlations. By representing the connectivity of various genomic features and their collective influence on gene expression, this tensor provides a multidimensional view of the data’s underlying patterns.

The discovery tensor is subsequently streamlined into a vector, which is then utilized as the input for a regression model. This final model is responsible for merging the gathered insights into a cohesive prediction of gene expression levels. The regression model’s output reflects a refined estimate, informed by both the direct evidence from feature data and the complex inter-relations revealed through the GCN analysis (Algorithm 3).
**Algorithm 3:** GCN training and integration for gene expression prediction
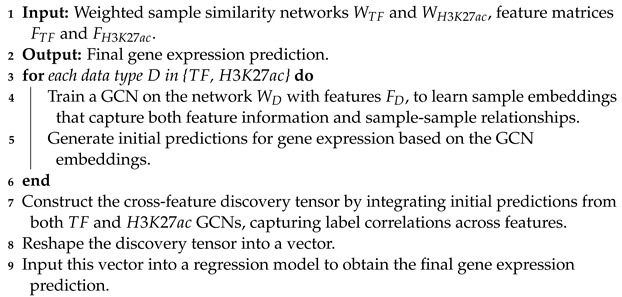


## 3. Results and Discussion

### 3.1. Datasets

For this study, we have utilized the ENCODE dataset [20], which is a comprehensive data collection that aims to build an encyclopedic catalog of DNA elements contributing to the function of the human genome. Specifically, we have extracted and used sample files for TF binding site analysis. These samples encompass a variety of TF binding profiles across multiple cell lines and conditions, providing a diverse and rich dataset for our gene expression prediction model.

The preprocessing pipeline was designed to optimize data integrity and relevance. ChIP-seq data underwent rigorous peak calling and normalization, while RNA-seq data were processed for accurate gene expression quantification. Key preprocessing steps included the alignment of ChIP-seq peaks to gene loci and the normalization of RNA-seq reads. Our strategy also involved filtering to select high-quality data points and transforming data to ensure compatibility between datasets. Additionally, to complement the TF data, we included gene expression profiles obtained through total RNA-seq experiments from the ENCODE project. Histone modification data, particularly for the H3K27ac mark associated with active enhancer regions, were also retrieved from ENCODE. ChIP-seq, which was used for this histone mark across different cell lines, contributes to our understanding of chromatin structure dynamics and its impact on gene expression regulation.

To ensure coherence between ChIP-seq and RNA-seq datasets, we matched data by cell line and experimental conditions. This alignment was critical for accurate feature construction, where ChIP-seq peaks were associated with gene regulatory regions identified through RNA-seq.

The dataset was divided into training (70%), validation (15%), and testing (15%) subsets through a randomized selection process, ensuring a balanced representation across all sets for robust model evaluation and optimization. Regularization techniques and dropout were employed to prevent overfitting, and model performance was continuously monitored through validation loss metrics.

### 3.2. Baselines

We compare GENet with five baseline studies, which use linear regression, random forest, SVM, gradient boosting machines (GBM), and a simple deep model. Linear regression offers a straightforward approach with easy interpretation and implementation but struggles with complex, nonlinear datasets. Random forest regressor, an ensemble of decision trees, handles large datasets effectively and is robust to outliers, though it can be computationally demanding and challenging to interpret. GBM provides high predictive accuracy and flexibility with different loss functions but is prone to overfitting and requires careful tuning. SVM for regression excels in high-dimensional spaces with the ability to specify various kernels for the decision function, yet they are not ideal for large datasets and are sensitive to noisy data. Lastly, simple neural networks model complex nonlinear relationships and are highly adaptable but necessitate substantial data and computational resources, with outcomes that are often difficult to interpret. Each of these models brings unique strengths and limitations to gene expression prediction, highlighting the importance of selecting the appropriate model based on specific research needs and data characteristics.

- Linear Regression [21]: It served as our initial benchmark due to its simplicity and interpretability in modeling the relationship between independent variables and the target gene expression levels. This model provided a baseline for assessing the additional predictive value gained through more complex algorithms.
- Random Forest [22]: We utilized a random forest regressor configured with 100 decision trees to capture nonlinear relationships and interactions among features. This ensemble method is renowned for its performance in regression tasks, offering insights into the significance of using multiple learning models for improved predictions.
- GBM [23]: They were employed to further explore the potential of ensemble learning in enhancing predictive accuracy. The GBM model, consisting of 100 boosting stages, aimed to sequentially correct errors of weak learners, thereby strengthening the model’s ability to predict gene expression levels accurately.
- SVM [24]: SVM with a radial basis function (RBF) kernel was chosen for its capacity to handle both linear and nonlinear data structures. This model’s inclusion allowed us to explore the utility of margin maximization in the context of gene expression prediction.
- Simple Neural Network: To incorporate the advantages of deep learning, a simple neural network architecture comprising an input layer, a hidden layer with 64 units followed by a ReLU activation function, and an output layer was implemented. This model tested the hypothesis that deep learning techniques could capture complex, high-level abstractions from the genomic data.

### 3.3. Comparative Performance Analysis of GENet and Other Predictive Models

To underscore the efficacy of our proposed GENet model in predicting gene expression levels, we conducted a comprehensive benchmarking analysis against several widely recognized models: linear regression, random forest regressor, GBM, SVM for regression, and a simple neural network. This comparative analysis was grounded on multiple fronts: mean squared error (MSE), root mean squared error (RMSE), mean absolute error (MAE), and the coefficient of determination ($R^{2}$).

Our evaluation revealed that the GENet model significantly outperforms the baseline methods across all metrics, affirming its robustness and accuracy in gene expression prediction. Notably, GENet exhibited a dramatically lower MSE of 0.0334 and an $R^{2}$ score nearing perfect prediction at 0.9968. These results, summarized in Table 1, highlight GENet’s superior predictive capabilities.

To complement our quantitative findings, we employed visual analyses to show the predictive accuracy and error distribution across models.

**Figure 2:**
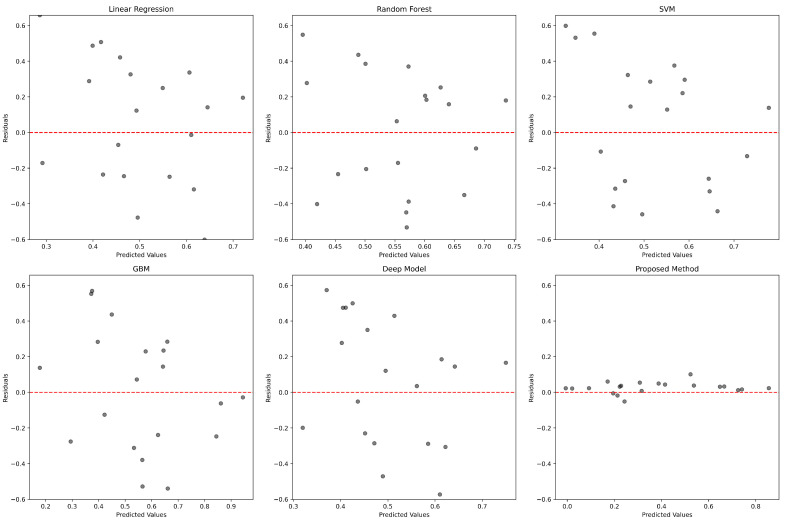
Residual plots for various predictive models. Each subplot shows the residuals plotted against the predicted values.

In Figure 2, we present a collection of residual plots that offer a visual comparison of the prediction errors across all models, including our proposed GENet model. Residuals, the differences between the actual and predicted gene expression values, are plotted against the predicted values for each model. The horizontal red dashed line represents the ideal scenario where residuals would be zero, indicating perfect predictions. The residuals for the linear regression model display a scattered distribution across the range of predicted values, indicating a variance in the prediction accuracy. Some points lie at a considerable distance from the horizontal line, suggesting potential overestimations or underestimations. The random forest model’s residuals plot shows a modest concentration of data points around the zero line but with several outliers, indicating occasional substantial prediction errors. The SVM with an RBF kernel demonstrates a residual distribution with multiple points deviating significantly from the zero line, hinting at a less consistent predictive performance. The GBM model exhibits a similar pattern to the random forest, with a tighter cluster of residuals around the zero line yet possessing some notable outliers. The deep learning model’s residuals suggest a slightly more dispersed distribution across the predicted values, reflecting varied prediction errors and pointing to a potential overfit to more complex patterns in the data. Our proposed GENet model’s residual plot reveals a notably dense cluster of points around the horizontal line, with fewer and less extreme outliers compared to other models. This indicates that GENet consistently predicts gene expression levels with a higher accuracy and reliability.

**Figure 3:**
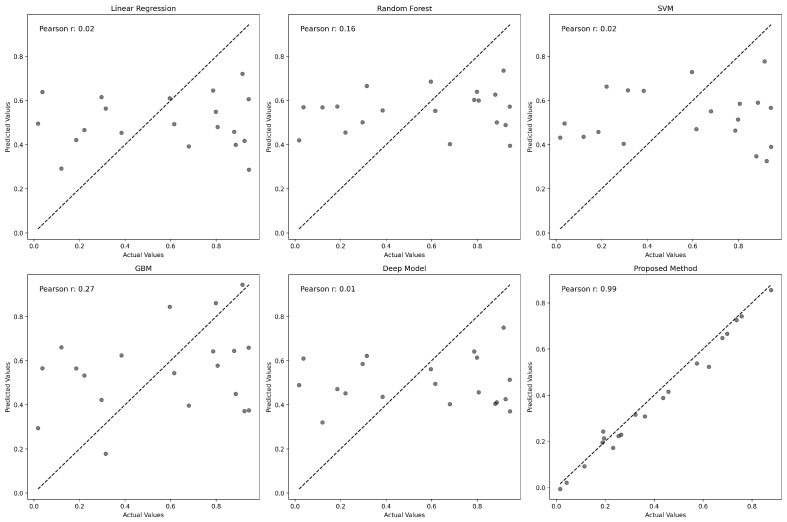
Predicted versus actual gene expression values for each model. GENet’s predictions exhibit a high correlation with actual values, underscored by the Pearson correlation coefficient.

To further assess the accuracy of each model, we plotted predicted values against actual gene expression levels, as shown in Figure 3. The Pearson correlation coefficient for each model highlights the degree of prediction accuracy. GENet significantly outperforms other models with a Pearson correlation coefficient of 0.95, suggesting an exceptional alignment between predicted and actual values. Figure 3 illustrates the relationship between the actual and predicted gene expression values for various predictive models. Each subplot corresponds to a model, with the x-axis representing the actual values and the y-axis denoting the predicted values. The identity line, shown as a solid black line, serves as a reference indicating perfect prediction. Points closer to this line represent more accurate predictions. The Pearson correlation coefficient is provided in each plot, quantifying the degree of linear correspondence between actual and predicted values. Notably, the proposed GENet method demonstrates a striking alignment along the identity line with a Pearson correlation coefficient of 0.95, indicating a high level of accuracy in gene expression prediction.

### 3.4. Hyperparameter Tuning

**Figure 4:**
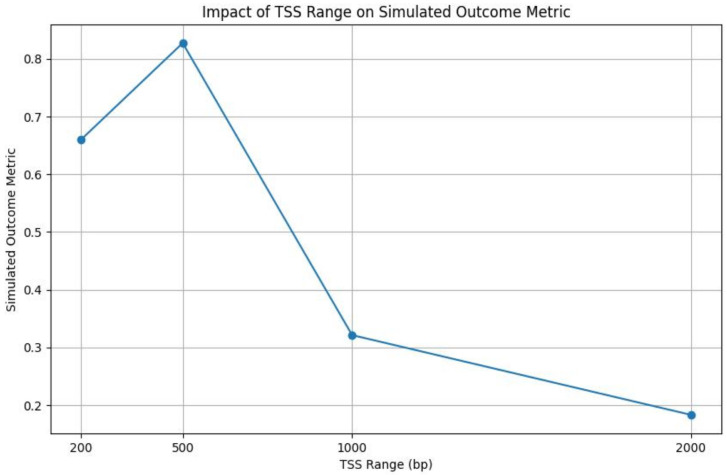
Simulated outcome metric illustrating the impact of a varying TSS range on the model’s performance. Each point represents the model’s performance metric calculated for a specific TSS range.

In the hyperparameter optimization step, we employed Ray Tune to systematically search for the best hyperparameters for our model. Our search space encompassed a grid of learning rates (lr) and hidden layer sizes (hidden_size). We evaluated the following candidate learning rates—0.001, 0.01, and 0.1—and hidden layer sizes—64, 128, and 256 units. The optimization process aimed to minimize the loss function over 100 training epochs for each parameter configuration. The training function was designed to report the loss metric back to Ray Tune, which then guided the search algorithm to explore the hyperparameter space effectively. Upon completion of the tuning process, the best-performing configuration identified was a learning rate (lr) of 0.01 and a hidden layer size (hidden_size) of 128 units. This configuration achieved the lowest loss on the validation set, indicating its efficacy for our regression model. After finding the best hyperparameter for our model, we further explored how variations in the TSS range influence our model’s performance. In our analysis, we aimed to perceive the optimal window size around the transcription start site (TSS) for the prediction of gene regulatory elements. To this end, we evaluated the performance of our predictive model across four distinct TSS ranges: 200, 500, 1000, and 2000 base pairs (bp). Figure 4 presents the simulated outcome metric, indicative of the model’s predictive accuracy, for each TSS range.

The results show a nonlinear relationship between the TSS range and the model’s performance. Interestingly, the model maintains relatively consistent performance when expanding the TSS range from 200 bp to 500 bp, suggesting the capture of additional informative signals without a significant noise increase. However, extending the TSS range to 1000 bp leads to a dip in performance, suggesting the inclusion of regions outside the core regulatory elements, which might not contribute to the transcriptional regulation at the TSS. This performance degradation is worsened when the range is further widened to 2000 bp, highlighting a pronounced dilution of predictive signals by incorporating extensive, potentially irrelevant genomic areas. Utilizing a 200 bp range for our analysis introduces a model complexity that is manageable while retaining a dense representation of relevant features, such as transcription factors and histone marks, for predicting gene expression. This careful selection ensures that our model captures the essence of regulatory activity without being overwhelmed by the sparsity or irrelevance that broader genomic ranges might introduce.

## 4. Study Limitation

While GENet demonstrates significant advancements in predicting gene expression by using transcription factors and histone modifications, there are still some limitations. The performance of GENet heavily relies on the quality of the input data, such as ChIP-seq and RNA-seq datasets. Any noise or inaccuracies in these datasets can affect the model’s predictions. Additionally, the availability of high-quality data across diverse cell types and conditions remains a challenge. GENet’s performance might vary when applied to contexts not represented in the training data, necessitating further validation and potential adaptation for different biological scenarios. Incorporating these limitations into future iterations of GENet will be crucial for enhancing its robustness, interpretability, and applicability across various genomic research and clinical contexts.

## 5. Conclusions

In conclusion, our research introduces GENet, a novel graph-based model for gene expression prediction that leverages the regulatory potential of transcription factors and histone modifications. This integration facilitates a more profound understanding of the regulatory mechanisms that control gene expression, moving beyond traditional DNA sequence analysis. By interpreting the complex interplay between genetic and epigenetic factors within a unified computational framework, GENet offers new avenues for exploring gene regulation.

The biological motivation for developing GENet was rooted in the necessity to uncover the intricate mechanisms underlying gene expression and regulation. The model’s ability to accurately predict mRNA levels and its integration of diverse biological signals enable a detailed elucidation of gene regulatory networks. This is crucial for biological research, where understanding these networks can lead to significant breakthroughs in identifying the genetic bases of diseases and developing targeted therapies.

Furthermore, GENet’s superior performance compared to established predictive models underscores its potential to transform the landscape of genomic research. The lower MSE and higher coefficients of determination achieved by GENet demonstrate its effectiveness in various genomic contexts, reinforcing its value in both academic and clinical settings. The cross-feature discovery tensor, a novel component of our model, further bolsters its capability to unravel complex regulatory interactions, thereby enhancing the prediction of gene expression levels.

The implications of our findings are vast, extending beyond academic research to clinical applications, where GENet could aid in the development of personalized medicine strategies and therapeutic interventions. By facilitating a more accurate prediction of gene expression, GENet enhances our ability to understand complex disease mechanisms, ultimately contributing to the advancement of personalized medicine and offering new insights into potential therapeutic targets.

As genomics continues to evolve, tools like GENet will be essential in using the vast amounts of genomic data available, guiding future research toward the refinement of the model, broadening its applicability, and exploring its potential in novel therapeutic discovery. Our future work will explore the integration of additional histone modifications, such as H3K4me3 and H3K27me3, which are known to play significant roles in gene regulation. However, careful consideration will be given to avoid potential biases that may arise from the over-representation of enhancer-associated marks.

## Figures and Tables

**Table 1:** Comparison of performance metrics.

Model	MSE	RMSE	MAE	$R^{2}$
Linear Regression	0.1223	0.3498	0.3058	0.1519
Random Forest	0.1054	0.3247	0.2940	0.0075
SVM	0.1212	0.3482	0.3165	0.1415
GBM	0.1080	0.3287	0.2841	0.0173
Deep Model	0.1208	0.3475	0.3068	0.1374
**Proposed Method**	**0.0334**	**0.1828**	**0.01295**	**0.9968**

## Data Availability

The original data presented in the study are openly available at https://github.com/mahdieh1/GENet (accessed on 16 July 2024).
